# Barriers to obstetric care at health facilities in sub-Saharan Africa - a systematic review protocol

**DOI:** 10.1186/s13643-015-0045-z

**Published:** 2015-04-23

**Authors:** Minerva Kyei-Nimakoh, Mary Carolan-Olah, Terence V McCann

**Affiliations:** College of Health and Biomedicine, Victoria University, PO Box 14428, Melbourne, Victoria 8001 Australia

**Keywords:** Obstetric care, Maternity care, Access, Barriers, Facility-based deliveries, Maternal deaths, Institutional maternal mortality, Sub-Saharan Africa, Developing countries, Systematic review

## Abstract

**Background:**

Since the launch of the Millennium Development Goals (MDGs) by the United Nations in 2000, the global community has intensified efforts to reduce adverse maternal health outcomes, especially, in sub-Saharan Africa. Despite these efforts, there is an increasing concern that the decline in maternal deaths has been less than optimal, even for women who receive birthing care in health facilities. High maternal deaths have been attributed to a variety of issues such as poor quality of care, inadequate resources, poor infrastructure, and inaccessibility to healthcare services. In other words, even in settings where they are available, many women do not receive life-saving obstetric care, when needed, despite the fact that basic and comprehensive obstetric care is widely recognized as a key to meeting maternal health goals. It is important to understand the common challenges that this developing region is facing in order to ensure a more rapid decline in adverse maternal health outcomes. The aim of this review is to synthesize literature on barriers to obstetric care at health institutions which focuses on sub-Saharan Africa, the region that is most affected by severe maternal morbidity and mortality.

**Methods:**

This review follows guidelines by the preferred reporting items for systematic reviews and meta-analyses (PRISMA) checklist. An electronic search of published literature will be conducted to identify studies which examined barriers to health facility-based obstetric care in sub-Saharan Africa. PubMed, Cumulative Index to Nursing and Allied Health Literature (CINAHL), and Scopus databases will be searched. Published articles in English, dated between 2000 and 2014, will be included. Combinations of search terms such as obstetric care, access, barriers, developing countries, and sub-Saharan Africa will be used to locate related articles, and eligible ones retained for data abstraction. A narrative synthesis approach will be employed to synthesize the evidence and explore relationships between included studies.

**Discussion:**

Information on the barriers to obstetric care is needed to inform policies for the improvement of maternal health. This review will contribute to providing related vital evidence to facilitate removal of barriers to maternal health services and interventions.

**Systematic review registration:**

PROSPERO 2014:CRD42014015549.

**Electronic supplementary material:**

The online version of this article (doi:10.1186/s13643-015-0045-z) contains supplementary material, which is available to authorized users.

## Background

Sub-Saharan Africa has the world’s highest adverse maternal health outcomes. The designation sub-Saharan Africa as employed in this paper refers to its usage as given in the United Nations (MDGs) regions’ groupings, where it is used to indicate all of Africa except northern Africa (that is, Algeria, Egypt, Libyan Arab Jamahiriya, Morocco, Tunisia, and Western Sahara) [1]. The lifetime risk of maternal death in sub-Saharan Africa is 1 in 38 compared to 1 in 160 for developing regions in general and 1 in 3,800 in developed regions [2]. Between 1990 and 2013, there has been a 45% decline in global maternal mortality ratio (MMR), that is, from 380 to 210 deaths per 100,000 live births. Despite this decline, sub-Saharan Africa had a high MMR of 510 per 100,000 live births, compared to Northern Africa which generally had an average MMR of 69 per 100,000 live births in 2013. As a consequence, sub-Saharan Africa accounted for 62% of global maternal deaths in 2013 [2].

Several sub-Saharan African countries have made significant progress in reducing MMR, but maternal death trends are variable. An estimated 15% (and possibly more) of all pregnant women in the world develop serious obstetric complications [3,4], most of which are treatable [4]. The majority of these complications occur during, before, or shortly after birth [5]. This knowledge about when most of these deaths occur provides an excellent window for interventions that could improve outcomes. In order to minimize related threats to life and improve outcomes for mother and child, skilled care in a supportive environment is essential [6,7]. This is supported by the World Health Organization’s recommendations on basic and comprehensive emergency obstetric care (EmOC), which outlines essential services, level of healthcare delivery, and related skilled attendants required for safe care [3].

However, most sub-Saharan African nations are faced with a diverse range of individual/household problems as well as health system challenges. Challenges may include sociocultural barriers [8], poor maternity referral systems [9-11], shortage of skilled health personnel [12], and poor transport infrastructure coupled with long distances to health facilities [13-15]. Ultimately, these problems impact on access to skilled care at birth, which is integral to improved obstetric outcomes.

Basic and comprehensive EmOC is often not equitably distributed in many sub-Saharan African countries, in terms of its availability, accessibility, and acceptability [5,16,17]. Additionally, countries with the worst maternal healthcare outcomes also have the least number of health workforce per population [7]. As reported in the 2014 *State of the World*’*s Midwifery Report*, 73 countries have 78% of births worldwide, 96% of global maternal deaths, and less than 42% of the world’s midwives, nurses, and doctors [7]. Not surprisingly, the rate of skilled care at birth in Africa is low, at about 51%, with considerable rural/urban and socio-economic disparities [18]. Even among those who receive skilled care at birth, adverse obstetric outcomes remain higher than expected. Given substantial efforts invested in encouraging women to use formal birthing services, end users may find obstetric care services more appealing if outcomes are significantly improved. In fact, institutional (health facility-based) maternal deaths and other adverse outcomes are significantly higher in developing regions, such as sub-Saharan Africa and South East Asia [5].

This situation points partly to challenges regarding access to timely and appropriate obstetric care within health facilities. Of equal significance is safeguarding the trust of healthcare service users in facility-based care, without which maternal health outcomes are only likely to worsen. This is important because skilled care in sub-Saharan Africa is generally available in health facilities. Nonetheless, a larger than average number of deaths occur in healthcare facilities. Institutional maternal deaths may occur before or after receiving obstetric care. The former may be accounted for by delays in seeking care by women/families or a poor referral system; and the latter raises concerns about the nature of care provided and possible challenges [5].

### Significance

In light of these maternal health challenges, this systematic review will focus on gathering evidence from peer-reviewed literature on barriers to timely and appropriate obstetric care in sub-Saharan Africa. Apart from potentially improving obstetric care received by women in health facilities, identifying and removing barriers in healthcare settings could ultimately help boost skilled care attendance. This is because observable improvement in maternity outcomes may be a strong motivation for other women to choose healthcare facilities for birthing services. In other words, given the pivotal role of basic and comprehensive EmOC, synthesis of related literature will provide evidence to help strengthen policies aimed at improving obstetric care practice and also facilitate efforts in promoting the use of birthing services. Considering the strategic role of skilled care in reducing maternal deaths and on-going efforts to encourage healthcare facility-based births, it is crucial to ensure that scarce resources allocated to these efforts yield intended outcomes. This review will help assess the extent, strength, and implications of evidence across countries in sub-Saharan Africa.

### Scope of the systematic review

This review aims to examine literature on barriers to obstetric care at health institutions in sub-Saharan Africa. It will focus on barriers or challenges that emerge after pregnant women have decided to seek obstetric care. It will also consider such barriers from the perspectives of maternity care workers (supply-side barriers) and service users (demand-side barriers) accessing formal maternity care services. Demand-side barriers are independent of service delivery or price and occur at the household and community level, such as transport costs to health facilities and lack of health awareness. Supply-side barriers, on the other hand, are constraints at the service delivery level and are beyond the control of health service users, such as long waiting times and high service costs [19]. Articles of interest will be quantitative studies targeting maternity care workers and pregnant women accessing care and addressing any of the following:

- barriers to accessing obstetric care and referral services,
- barriers/challenges to receiving timely and appropriate care while utilizing maternity services, and
- barriers encountered by maternity care workers in providing obstetric care and referral services.

## Methods

### Data sources

We will search the online databases PubMed, (CINAHL), and Scopus. Reference lists from located papers will also be checked and papers assessed for eligibility.

### Search strategy

The search will be conducted using combinations of the search term ‘obstetric care’ with ‘access,’ ‘barriers,’ ‘developing countries,’ ‘pregnancy,’ ‘morbidity,’ ‘mortality,’ ‘hemorrhage,’ ‘hypertensive disorders of pregnancy,’ ‘sepsis/infection,’ ‘obstructed labor,’ ‘abortion-related complications,’ and ‘sub-Saharan Africa’ to locate relevant articles. Also, a combination of the key terms with each individual country of the region will be used. Based on eligibility terms developed in consultation with experts, relevant studies published in English, between 2000 and 2014, will be retrieved. The year 2000 was selected as a starting point because that was when the MDGs were launched and many developing countries began tracking maternal health issues more closely. The search strategy for this review will be as follows:

1. Journals indexed in PubMed, CINAHL, and Scopus will be extensively searched using pre-identified key search terms and relevant synonyms.
2. A preliminary screening of articles in the search results will be conducted by checking the titles and abstracts, in order to appraise their eligibility.
3. Potentially relevant articles will be extracted for further examination, and those that do not meet the eligibility criteria will be excluded.
4. Reference lists of retrieved articles will be searched for additional papers and possible inclusion.
5. Full texts of all studies meeting the inclusion criteria will be retained for detailed review and analysis.

### Eligibility criteria

The following criteria will guide data abstraction.

### Inclusion criteria

1. Peer-reviewed studies which report barriers to accessing, receiving, or providing obstetric care services at healthcare facilities (from the perspectives of service users and maternity caregivers) will be included. Barriers will be limited to those encountered after the decision to seek formal maternity care services has been made by pregnant women.
2. Only studies using quantitative methods will be considered, provided the study was conducted in sub-Saharan Africa.

### Exclusion criterion

1. Studies must have been published in English and report results of obstetric care barriers between 2000 and 2014.

### Selection of studies

We will assess studies for possible inclusion using the criteria outlined above by an initial screening of titles and abstracts. The review will consider quantitative studies conducted in community and hospital settings covering research objectives that coincide with those within the scope of this review.

### Quality assessment

This review is guided by the preferred reporting items for systematic reviews and meta-analyses (PRISMA) checklist by Moher D. *et al.* [20]. As a measure of quality, two investigators will independently review all full-text articles deemed eligible. If any differences arise, discussions will continue until consensus is reached. Quality of selected studies will be assessed using the Quality Assessment Tool for Quantitative Studies by the Effective Public Health Practice Project (EPHPP ) [21,22] (See Additional file 1). Assessment of individual studies for methodological quality is an essential step in systematic reviews, as it imposes some rigor in minimizing bias. The Quality Assessment Tool for Quantitative Studies by the EPHPP [21] was considered as an appraisal tool for this review as it is useful for the assessment of clinical and observational studies. Its components are also consistent with the checklist of items outlined by the Strengthening the Reporting of Observational Studies in Epidemiology (STROBE) Statement [23]. Based on an initial scoping exercise, the types of studies which will be included in this review are mostly observational, such as cross-sectional and cohort studies. The EPHPP tool assesses internal and external validity of such studies and has been demonstrated to yield excellent inter-rater agreement [24], as well as acceptable content and construct validity [22]. Compared to the Cochrane Collaboration Risk of Bias Tool, EPHPP’s tool was found to perform better on inter-rater agreement for individual domains and inter-rater agreement for the final grade [24]. The tool was developed for use in systematic reviews of public health interventions and has since been widely cited in several published reviews [25-27]. The tool is accompanied by a supplemental document (Quality Assessment Tool for Quantitative Studies Dictionary) which explains the terms in the instrument and provides clear guidance on how to grade eligible studies [28]. The EPHPP Quality Assessment Tool assesses six domains which include selection bias, study design, confounders, blinding, data collection methods and withdrawals, and dropouts. The tool ranks methodological quality for each component, and then globally. Based on the rating of each component, an overall quality rating of weak, moderate, or strong is assigned to the study under review. Studies are categorized as ‘strong’ if it receives no weak rating, ‘moderate,’ if given one weak rating, or ‘weak’ if given two or more weak ratings. The tool also includes two other domains, which are intervention integrity and analyses. These additional elements are assessed but not graded. At the end of the quality assessment process, reviewers will discuss and reach a consensus on results which will improve inter-rater reliability.

### Data abstraction

We will abstract data from retained articles using a data abstraction form (See Additional file 2). Data will include publication information such as author, journal, year of publication, and location of study. The study design used will be noted. Specific details on the study, such as sample size, response rate, and population characteristics, will also be captured. Data on phenomena of interest reported as barriers/challenges to obstetric care will be collected.

### Synthesis of data

Considering the nature and likely diversity of the outcomes, summary of data will be carried out using narrative synthesis of the barriers to obstetric care. In order to ensure a robust and transparent synthesis of the evidence, the guidance on the conduct of narrative synthesis in systematic reviews by Popay J. H. *et al.* [29] will be used in conducting the narrative synthesis. The guidance offers four elements, along with several related tools and techniques that may be applied in the synthesis process. The four elements include the following:

1. developing a theory,
2. developing a preliminary synthesis,
3. exploring relationships within and between studies, and
4. assessing the robustness of the synthesis.

The preliminary plan for this review is explained below.

1. Developing a theory: The basic theory underlying the review is that by identifying barriers from both service user and provider viewpoints, policymakers and healthcare workers can match expectations from both sides to enhance maternity care access. Jacobs and colleagues’ [30] analytical framework regarding barriers to healthcare is of particular interest and will later form the basis of comparison across the studies. The framework is based on four broad categories (that is, geographic accessibility, availability, affordability, acceptability). Under these broad categories, the barriers are further grouped as supply-side or demand-side. Where theories underlying the work are described in primary studies, these will be included in the discussion to facilitate interpretation of their findings.
2. Developing a preliminary synthesis: The tools/techniques selected for application include drawing of tables, groupings and clusters, textual descriptions of studies, and transforming data into a common rubric. This stage of the process will set the stage for further analysis. Due to possible wide variations in the studies, they will be laid out in tables to provide an initial overview of the relationship between the studies. The table(s) may be organized according to the setting/context (community or health facility-based), type of subjects involved (health workers or service users), study design, results of study quality assessment, and outcome measures. Since the review broadly examines two different populations (health service users and providers), studies will naturally be sorted into these clusters in order to facilitate comparison and interpretation. Any other groupings will be dependent on the nature of data extracted, as reflected in the table(s). Subsequently, textual descriptions will help draw out and report vital aspects of the studies shown in the table(s). Summary tables using crude data on barriers will be generated from included papers. Where possible, results of studies that have undertaken significance testing will be summarized and pooled to arrive at a common statistic. In order to assess effects, statistical measures such as odds ratio will also be computed.
3. Exploring relationships within and between studies: The tools/techniques which will be of value at this stage are sub-group analyses and qualitative case descriptions. The processes described above will help assess similarities and differences between studies. Differences in obstetric care barriers will be discussed by location, type of facility, types of maternity care workers, and other relevant sub-categories. By comparing and contrasting, we will explore how factors such as the study design, population characteristics, and context may explain the study results. The analytical framework [30] on selecting appropriate interventions for barriers to health services will further contribute to sub-group analysis at this stage of the narrative synthesis process. Building upon the initial textual descriptions, vital aspects of the included studies will be qualitatively described more comprehensively and interpreted to enhance understanding of any discrepancies between studies.
4. Assessing the robustness of the synthesis: Validity assessment and critical reflection on the synthesis process will be employed. As explained above, assessment of the methodological quality of the primary studies included in the review forms part of the data extraction process and therefore occurs at an earlier stage. Additionally, the narrative synthesis process will be critically reflected upon. At the end of the review, the exact process applied will be reported in the final paper.

## Discussion

In this review, we will conduct a critical appraisal of literature on barriers to obstetric care in sub-Saharan Africa in order to make comparisons across countries in the last 14 years. As the deadline for meeting the MDGs is approaching, it is becoming more apparent that the sub-Saharan Africa region will be unlikely to meet set targets. The findings will contribute to greater understanding of challenges in providing obstetric care and also offer evidence needed to improve maternal health outcomes in a region, where the need for such evidence is greatest.

## Additional files

Additional file 1:
**Effective Public Health Practice Project (EPHPP) Quality Assessment Tool for Quantitative Studies.**
Additional file 2:
**Data abstraction form.** Description of items included in the abstraction of data from eligible studies.

## Abbreviations

EmOC: basic and comprehensive emergency obstetric care
EPHPP: Effective Public Health Practice Project
MDGs: Millennium Development Goals
MMR: maternal mortality ratio
PRISMA: preferred reporting items for systematic reviews and meta-analyses
STROBE: Strengthening the Reporting of Observational Studies in Epidemiology
