# Supplementary material for: Barriers to obstetric care at health facilities in sub-Saharan Africa - a systematic review protocol
Source: Syst Rev. 2015 Apr 23;4:54. doi: 10.1186/s13643-015-0045-z (PMC4411746; doi:10.1186/s13643-015-0045-z)
Supplement: Additional file 1: — Effective Public Health Practice Project (EPHPP) Quality Assessment Tool for Quantitative Studies. [file 13643_2015_45_MOESM1_ESM.doc]

**Additional file 1: Quality Assessment Tool**

**EPHPP Quality Assessment Tool for Quantitative Studies**

**Reviewer:__________________ Author:_________________ Year:________**

| **A) SELECTION BIAS Applicable [ ] Not applicable [ ] (Move to Section B)** | | | | | | | | | | | | | | | | | | | | | | | |
| --- | --- | --- | --- | --- | --- | --- | --- | --- | --- | --- | --- | --- | --- | --- | --- | --- | --- | --- | --- | --- | --- | --- | --- |
| (Q1) Are the individuals selected to participate in the study likely to be representative of the target population? | 1 Very likely | | | | | | 2 Somewhat likely | | | | | | | | | 3 Not likely | | | | | | | 4 Can’t tell |
| (Q2) What percentage of selected individuals agreed to participate? | 1 80 - 100% agreement | | 2 60 – 79% agreement | | | | | | 3 less than 60% agreement | | | | | | | 4 Not applicable | | | | | | | 5 Can’t tell |
| **SECTION RATING** | **STRONG 1** | | | **MODERATE 2** | | | | | | | | | | | | | **WEAK 3** | | | | | | |
| **B) STUDY DESIGN Applicable [ ] Not applicable [ ] (Move to Section C)** | | | | | | | | | | | | | | | | | | | | | | | |
| Indicate the study design | 1 Randomized controlled trial  2 Controlled clinical trial  3 Cohort analytic (two group pre + post)  4 Case-control  5 Cohort (one group pre + post (before and after))  6 Interrupted time series  7 Cross-sectional  8 Population  7 Other specify ____________________________  8 Can’t tell | | | | | | | | | | | | | | | | | | | | | | |
| Was the study described as randomized? If NO, go to Component C. | No | | | | | | | | | Yes | | | | | | | | | | | | | |
| If Yes, was the method of randomization described? | No | | | | | | | | | Yes | | | | | | | | | | | | | |
| If Yes, was the method appropriate? | No | | | | | | | | | Yes | | | | | | | | | | | | | |
| **SECTION RATING** | **STRONG 1** | | | **MODERATE 2** | | | | | | | | | | | **WEAK 3** | | | | | | | | |
| **C) CONFOUNDERS Applicable [ ] Not applicable [ ] (Move to Section D)** | | | | | | | | | | | | | | | | | | | | | | | |
| (Q1) Were there important differences between groups prior to the intervention? | 1 Yes | | | 2 No | | | | | | | | | | | 3 Can’t tell | | | | | | | | |
| (Q2) If yes, indicate the percentage of relevant confounders that were controlled (either in the design (e.g. stratification, matching) or analysis)? | 1 80 – 100% (most) | | | | | 2 60 – 79% (some) | | | | | | | 3 Less than 60% (few or none) | | | | | | | | | 4 Can’t Tell | |
| **SECTION RATING** | **STRONG 1** | | | **MODERATE 2** | | | | | | | | | | | **WEAK 3** | | | | | | | | |
| **D) BLINDING Applicable [ ] Not applicable [ ] (Move to Section E)** | | | | | | | | | | | | | | | | | | | | | | | |
| (Q1) Was (were) the outcome assessor(s) aware of the intervention or exposure status of participants? | 1 Yes | | | | | 2 No | | | | | | | | | 3 Can’t tell | | | | | | | | |
| (Q2) Were the study participants aware of the research question? | 1 Yes | | | | | 2 No | | | | | | | | | 3 Can’t tell | | | | | | | | |
| **SECTION RATING** | **STRONG 1** | | | | | **MODERATE 2** | | | | | | | | | **WEAK 3** | | | | | | | | |
| **E) DATA COLLECTION METHODS Applicable [ ] Not applicable [ ] (Move to Section F)** | | | | | | | | | | | | | | | | | | | | | | | |
| (Q1) Were data collection tools shown to be valid? | 1 Yes | | | | | 2 No | | | | | | | | | | 3 Can’t tell | | | | | | | |
| (Q2) Were data collection tools shown to be reliable? | 1 Yes | | | | | 2 No | | | | | | | | | | 3 Can’t tell | | | | | | | |
| **SECTION RATING** | **STRONG 1** | | | | | **MODERATE 2** | | | | | | | | | | **WEAK 3** | | | | | | | |
| **F) WITHDRAWALS AND DROP-OUTS Applicable [ ] Not applicable [ ] (Move to Section G)** | | | | | | | | | | | | | | | | | | | | | | | |
| (Q1) Were withdrawals and drop-outs reported in terms of numbers and/or reasons per group? | 1 Yes | 2 No | | | | | | 3 Can’t tell | | | | | | 4 Not Applicable (i.e. one time surveys or interviews) | | | | | | | | | |
| (Q2) Indicate the percentage of participants completing the study. (If the percentage differs by groups, record the lowest). | 1 80 - 100% | 2 60 - 79% | | | | | | 3 less than 60% | | | | | | 4 Can’t tell | | | | | | 5 Not Applicable (i.e. Retrospective case-control) | | | |
| **SECTION RATING** | **STRONG 1** | | | | | **MODERATE 2** | | | | | | | | | | | | **WEAK 3** | | | | | |
| **G) INTERVENTION INTEGRITY Applicable [ ] Not applicable [ ] (Move to Section H)** | | | | | | | | | | | | | | | | | | | | | | | |
| (Q1) What percentage of participants received the allocated intervention or exposure of interest? | 1 80 -100% | | | | 2 60 - 79% | | | | | | | 3 less than 60% | | | | | | | 4 Can’t tell | | | | |
| (Q2) Was the consistency of the intervention measured? | 1 Yes | | | | 2 No | | | | | | | | | | | | 3 Can’t tell | | | | | | |
| (Q3) Is it likely that subjects received an unintended intervention (contamination or co-intervention) that may influence the results? | 4 Yes | | | | 5 No | | | | | | | | | | | | 6 Can’t tell | | | | | | |
| **H) ANALYSES Applicable [ ] Not applicable [ ]** | | | | | | | | | | | | | | | | | | | | | | | |
| (Q1) Indicate the unit of allocation | community organization/institution | | | | | | | | | | practice/office | | | | | | | | | | individual | | |
| (Q2) Indicate the unit of analysis | community organization/institution | | | | | | | | | | practice/office | | | | | | | | | | individual | | |
| (Q3) Are the statistical methods appropriate for the study design? | 1 Yes | | | | | | | | | | 2 No | | | | | | | | | | 3 Can’t tell | | |
| (Q4) Is the analysis performed by intervention allocation status (i.e. intention to treat) rather than the actual intervention received? | 1 Yes | | | | | | | | | | 2 No | | | | | | | | | | 3 Can’t tell | | |

**SUMMARY OF COMPONENT RATINGS**

**Please transcribe the information from the section ratings onto the table below**.

| **A** | **SELECTION BIAS** | Strong | Moderate | Weak |  |
| --- | --- | --- | --- | --- | --- |
|  |  | **1** | **2** | **3** | Not Applicable |
| **B** | **STUDY DESIGN** | Strong | Moderate | Weak |  |
|  |  | **1** | **2** | **3** | Not Applicable |
| **C** | **CONFOUNDER** | Strong | Moderate | Weak |  |
|  |  | **1** | **2** | **3** | Not Applicable |
| **D** | **BLINDING** | Strong | Moderate | Weak |  |
|  |  | **1** | **2** | **3** | Not Applicable |
| **E** | **DATA COLLECTION METHODS** | Strong | Moderate | Weak |  |
|  |  | **1** | **2** | **3** | Not Applicable |
| **F** | **WITHDRAWALS AND DROPOUTS** | Strong | Moderate | Weak |  |
|  |  | **1** | **2** | **3** | Not Applicable |

**G ANALYSIS**

Comments

___________________________________________________________________________

___________________________________________________________________________

**H INTERVENTION INTEGRITY**

Comments

___________________________________________________________________________

__________________________________________________________________________

**Global rating for individual paper (circle one):**

**1 STRONG** (no WEAK ratings)

**2 MODERATE** (one WEAK rating)

**3 WEAK** (two or more WEAK ratings)

Is there a discrepancy between the two reviewers with respect to the component (A-F) ratings?

No Yes

If yes, indicate the reason for the discrepancy

1 Oversight

2 Differences in interpretation of criteria

3 Differences in interpretation of study

Final decision of both reviewers (circle one):

**1 STRONG**

**2 MODERATE**

**3 WEAK**
