# Supplementary material for: Barriers to obstetric care at health facilities in sub-Saharan Africa - a systematic review protocol
Source: Syst Rev. 2015 Apr 23;4:54. doi: 10.1186/s13643-015-0045-z (PMC4411746; doi:10.1186/s13643-015-0045-z)
Supplement: Additional file 2: — Data abstraction form. Description of items included in the abstraction of data from eligible studies. [file 13643_2015_45_MOESM2_ESM.doc]

**Additional file 2: Data Abstraction Form**

| **Part 1: Reviewer** |  |  |
| --- | --- | --- |

| **Part 2: Article details** | Author: | Year: |
| --- | --- | --- |
|  | Journal: | |

| **Part 3: Eligibility of article** | | | |
| --- | --- | --- | --- |
| 1. | Quantitative study conducted in sub-Saharan Africa and published in English between 2000 and 2014 | Yes | No |
| 2. | Study is health facility or community-based, reporting barriers to formal obstetric care | Yes | No |

| **Part 4: Study Setting** | | | | |
| --- | --- | --- | --- | --- |
| Article Title: | | | | |
| Article source: | | Author(s) | Journal | Year |
|  |  |  |
| 1. | Country (and sub-region): |  | | |
| 2. | What types (level) of health facilities were included? |  | | |
| 3. | What is the type of locality (rural/urban) |  | | |
| 4. | Where the facilities public/private? |  | | |
| 5. | Is the healthcare financing system (insurance/out of pocket) mentioned? |  | | |
| 6. | What types of maternity care providers attended the facility? |  | | |
| 7. | Where the skill mix in the facility appropriate to cope with the patient flow and case mix received? |  | | |
| 8. | Where there trained health providers present at the facility at all times (24 hours/day)? |  | | |
| 9. | What designated maternity care services (basic/comprehensive EmOC) did the facility provide? |  | | |
| 10. | Were the maternity wards adequately equipped to offer designated maternity care services? |  | | |
| 11. | Did the facility provide caesarean section services? |  | | |
| 12. | If applicable, was the operating theatre in good repair and fully equipped with drugs and surgical equipment to perform life-saving procedures, when required? |  | | |
| 13. | Did the facility provide post-abortion care? |  | | |
| 14. | Did the facility provide transfusion services? |  | | |
| 15. | Did the facility have a system for routinely reviewing maternal deaths or "near-miss deaths"? |  | | |
| 16. | Did the facility have a procedure for transporting women to another facility (if necessary), in an obstetric emergency? |  | | |

| **Part 5(a): Participant Characteristics (maternity care workers)** | | | | | |
| --- | --- | --- | --- | --- | --- |
| 1. | Age range: | | | | |
| 2. | Education: | | | | |
|  | Categories: |  |  | |  |
|  |  |  |  | |  |
|  |  |  |  | |  |
| 3. | professional/technical qualifications: | | | | |
|  | Categories: |  |  | |  |
|  |  |  |  | |  |
|  |  |  |  | |  |
| 4. | Years of experience: | | | | |
| **Part 5(b): Participant Characteristics (Service users)** | | | | | |
| 1. | Age | | |  | |
| 2. | Gestational Age | | |  | |
| 3. | Parity | | |  | |
| 4. | Nationality/Ethnic background | | |  | |
| 5. | Education | | |  | |
| 6. | Income levels (socioeconomic status) | | |  | |
| 7. | Family structure/ marital status | | |  | |

| **Part 6: Reported Outcomes** | | | | | |
| --- | --- | --- | --- | --- | --- |
| 1. | Maternal mortality | Yes | | No | |
|  | Rates: |  | | | |
|  | Causes: |  | | | |
|  |  |  | | | |
| 2. | Severe maternal morbidity | Yes | | No | |
|  | Categories: | Rates: | | | |
|  |  |  | | | |
|  |  |  | | | |
|  |  |  | | | |
| 3. | Neonatal mortality | Yes | | | No |
|  | Rates: | | | | |
| 4. | Neonatal morbidity | Yes | | | No |
|  | Categories: | | Rates: | | |
|  |  | |  | | |
|  |  | |  | | |

| **Part 7: Description of included studies** | | | | | | | | | | | | | | | |
| --- | --- | --- | --- | --- | --- | --- | --- | --- | --- | --- | --- | --- | --- | --- | --- |
| **Author, year and country of study** | | **Study design** | **Population and sample size** | | **Outcome variables** | **Reported barriers** | | | | | | | | | |
|  |  |  |  |  |  | **Service Users (Supply-side)** | | | | | **Maternity Care Workers (Demand-side)** | | | | |
|  | | | | | | **Geographic accessibility** | **Availability** | **Affordability** | **Acceptability** | **Other** | **Geographic accessibility** | **Availability** | **Affordability** | **Acceptability** | **Other** |
| 1. |  |  | |  |  |  |  |  |  |  |  |  |  |  |  |
| 2. |  |  | |  |  |  |  |  |  |  |  |  |  |  |  |
| 3. |  |  | |  |  |  |  |  |  |  |  |  |  |  |  |
| 4. |  |  | |  |  |  |  |  |  |  |  |  |  |  |  |
| 5. |  |  | |  |  |  |  |  |  |  |  |  |  |  |  |
| 6. |  |  | |  |  |  |  |  |  |  |  |  |  |  |  |
| 7. |  |  | |  |  |  |  |  |  |  |  |  |  |  |  |
| 8. |  |  | |  |  |  |  |  |  |  |  |  |  |  |  |
| 9. |  |  | |  |  |  |  |  |  |  |  |  |  |  |  |
| 10. |  |  | |  |  |  |  |  |  |  |  |  |  |  |  |

| **Part 8: Other Information/Comments** | | | | |
| --- | --- | --- | --- | --- |
| 1. | Funding source |  | | |
| 2. | Key conclusions |  | | |
| 3. | Clarification required | | Yes | No |
|  | If yes, list items that require clarification: | | | |
| 4. | Any other relevant comment(s) |  | | |
